# Potassium Citrate Supplementation Decreases the Biochemical Markers of Bone Loss in a Group of Osteopenic Women: The Results of a Randomized, Double-Blind, Placebo-Controlled Pilot Study

**DOI:** 10.3390/nu10091293

**Published:** 2018-09-12

**Authors:** Donatella Granchi, Renata Caudarella, Claudio Ripamonti, Paolo Spinnato, Alberto Bazzocchi, Annamaria Massa, Nicola Baldini

**Affiliations:** 1 Laboratory for Orthopedic Pathophysiology and Regenerative Medicine, IRCCS Istituto Ortopedico Rizzoli, via di Barbiano 1/10, 40136 Bologna, Italy; annamaria.massa88@gmail.com (A.M.); nicola.baldini@ior.it (N.B.); 2 Villalba Hospital, GVM Care and Research, Via di Roncrio, 25, 40136 Bologna, Italy; renata.caudarella@alice.it; 3 Osteoporosis and Metabolic Bone Disease Unit, IRCCS Istituto Ortopedico Rizzoli, via Pupilli 1, 40136 Bologna, Italy; claudio.ripamonti@ior.it; 4 Diagnostic and Interventional Radiology, IRCCS Istituto Ortopedico Rizzoli, via Pupilli 1, 40136 Bologna, Italy; paolo.spinnato@ior.it (P.S.); alberto.bazzocchi@ior.it (A.B.); 5 Department of Biomedical and Neuromotor Sciences, Via Pupilli 1, University of Bologna, 40136 Bologna, Italy

**Keywords:** osteopenia, potassium citrate, acid-base, urolithiasis, bone remodeling, bone turnover markers

## Abstract

The relationship involving acid-base imbalance, mineral metabolism and bone health status has previously been reported but the efficacy of the alkalizing supplementation in targeting acid overload and preventing bone loss has not yet been fully elucidated. In this randomized, double-blind, placebo-controlled study, the hypothesis that potassium citrate (K citrate) modifies bone turnover in women with postmenopausal osteopenia was tested. Three hundred and ten women were screened; 40 women met the inclusion criteria and were randomly assigned to the treatment or the placebo group. They were treated with K citrate (30 mEq day^−1^) or a placebo in addition to calcium carbonate (500 mg day^−1^) and vitamin D (400 IU day^−1^). At baseline and time points of 3 and 6 months, serum indicators of renal function, electrolytes, calciotropic hormones, serum bone turnover markers (BTMs) (tartrate-resistant acid phosphatase 5b (TRACP5b), carboxy-terminal telopeptide of type I collagen (CTX), bone alkaline phosphatase (BAP), procollagen type 1 N terminal propeptide (PINP)), and urine pH, electrolytes, and citrate were measured. The follow-up was completed by 17/20 patients in the “K citrate” group and 18/20 patients in the “placebo” group. At baseline, 90% of the patients exhibited low potassium excretion in 24 h urine samples, and 85% of cases had at least one urine parameter associated with low-grade acidosis (low pH, low citrate excretion). After treatment, CTX and BAP decreased significantly in both groups, but subjects with evidence of low-grade acidosis gained significant benefits from the treatment compared to the placebo. In patients with low 24h-citrate excretion at baseline, a 30% mean decrease in BAP and CTX was observed at 6 months. A significant reduction was also evident when low citrate (BAP: −25%; CTX: −35%) and a low pH (BAP: −25%; CTX: −30%) were found in fasting-morning urine. In conclusion, our results suggested that K citrate supplementation improved the beneficial effects of calcium and vitamin D in osteopenic women with a documented potassium and citrate deficit, and a metabolic profile consistent with low-grade acidosis.

## 1. Introduction

Bone mass tends to peak around the third decade of life, after which a continuous decline of 0.7% per year is considered to be physiologic. After menopause, however, bone loss is accelerated and, on average, women lose approximately 5% of trabecular bone and 1.5% of the total bone mass per year [1]. Postmenopausal estrogen deficiency is considered to be the major cause of accelerated bone loss [2]. It is associated with an increased rate of bone turnover, with an elevated number of active bone remodeling units (BRUs) in which the removal of bone by osteoclasts (OCs) prevails over the formation of bone matrix by osteoblasts (OBs) [3,4]. The imbalance between osteogenesis and bone resorption leads first to osteopenia and, subsequently, to osteoporosis, both characterized by decreased bone mineral density (BMD) (T-scores of <−1 and <−2.5, respectively), deterioration of bone microarchitecture, impairment of bone strength and, ultimately, increased susceptibility to fracture [5]. According to the “International Osteoporosis Foundation”, it is estimated that osteoporosis affects 200 million women worldwide, and the lifetime risk of experiencing fragility fractures to the distal wrist, vertebral body or proximal femur is approximately 30–35% for women over 50 years of age [6]. Disability due to osteoporotic fractures notably compromises the activities of daily living (ADL). Actually, the level of self-sufficiency is significantly decreased in 50% of women with hip fractures, and approximately 20% are candidates for long-term institutionalization [6]. Moreover, the risk of mortality in patients with fractures of the proximal femur is at least double with respect to an age-matched population [7]. While the highest rate of fractures is found in osteoporotic women, the absolute number is higher in osteopenic women due to the number of subjects with this condition [8]. This suggests that the risk profile of osteopenic women should be carefully evaluated shortly after menopause in order to identify the multiple factors which may promote bone frailty and make a “fracture event” more likely [8,9]. The risk of fracture is commonly evaluated by algorithms, i.e., the FRAX^®^ tool (https://www.sheffield.ac.uk/FRAX/) [10]. However, the evaluation of additional risk factors could be useful for identifying osteopenic women who may benefit from specific measures aimed at preventing bone loss.

Among the risk factors, it is well recognized that the proper intake of calcium and vitamin D plays an important role in preserving bone mass, but there are also other factors which seem to influence BMD [2]. For instance, adverse metabolic conditions (e.g., a high acid load) may increase the rate of bone loss and favor the onset and progression of osteopenia [11]. In fact, the skeleton is an alkaline reservoir which plays a pivotal role in maintaining the acid-base balance since it is able to modify the composition of the bone mineral matrix by acting as an “ion exchange column” and recovering alkali groups from hydroxyapatite in order to neutralize the proton excess [12]. In addition, it has been proven that acidosis directly influences the OC and OB activity. In fact, while OCs are virtually inactive at a pH of 7.4, the resorbing properties are stimulated when the pH drops below 6.9. The acidic milieu also significantly inhibits the osteogenic function, including the production of extracellular matrix, the activity of alkaline phosphatase, and the formation of trabecular bone [12].

A chronic subclinical inflammatory status [13], diets rich in salt and meat protein [14] as well as decreased renal function due to aging [15] are some of the conditions which determine low-grade acidosis. This is a metabolic condition in which the high acid load may exceed the physiological neutralization capacity while maintaining the blood pH within the normal range (7.35–7.45), albeit at values close to the lower limit [14]. While the nutritional hypothesis has been amended in systematic reviews and meta-analyses [11,16,17], the relationship between renal function, and the risk of osteoporosis or hip fracture has been confirmed in many studies. Recent findings have demonstrated that increased acid excretion [18], dysregulation of calcium homeostasis [19] and a decrease in urinary citrate [20,21,22] are recognizable in patients who exhibit osteopenia/osteoporosis and high bone turnover, thus, suggesting that urinary metabolic profiling may have significant implications for monitoring bone health status.

Since low pH is a risk factor which accelerates bone loss, some authors have proposed using strategies capable of opposing the acid overload as measures for preventing osteopenia progression or for supporting the treatment of osteoporosis. Proton pump inhibitors could be an effective strategy since these drugs have been designed specifically for counteracting proton excess [23]. However, they are not recommended for long-term treatment as they seem to enhance fracture risk by inhibiting calcium absorption [24]. Alternatively, alkalizing supplements have been proposed as a valid tool for limiting the adverse effects of acidosis and delaying bone loss [25]. For instance, potassium citrate (K citrate) is an alkaline compound used to increase urinary pH and prevent the precipitation of solutes in the urinary tract [26]. Previous in vitro studies demonstrated that K citrate is capable of preventing the adverse biological effects which a low extracellular pH induces on human bone cells [27]. A recent meta-analysis has shown that the daily administration of alkalizing supplements, including K citrate, for a time span ranging from 6 months to 2 years, led to a significant reduction in acid and calcium excretion, and a reduction in bone resorption, thus, suggesting a potential benefit to bone health [25]. However, the authors highlighted the significant heterogeneity of the studies, as the dosages, duration of treatment, patient inclusion criteria, primary outcomes and endpoints were very different. As a consequence, valid evidence in support of beginning the use of K citrate supplementation is still lacking.

To test the hypothesis that the daily administration of K citrate would be able to modify serum markers of bone resorption and formation, we performed a six-month prospective study in a homogeneous cohort of women with postmenopausal osteopenia. Moreover, laboratory parameters which could be useful in recognizing the osteopenic women who may benefit from the alkalizing treatment were searched for.

## 2. Materials and Methods

### 2.1. Study Design and Participants

A randomized, double-blind, placebo-controlled, parallel-group study, with balanced randomization (1:1) was designed. The study was approved by the local research committee and registered at Clinical Trial Gov (ID: NCT02731820). The participants were recruited from a cohort of postmenopausal women treated at the Radiodiagnostic Unit of Rizzoli Orthopedic Institute (IOR) from January 2016 to June 2017; periodic measurements of lumbar (at the L2–L4 level) and femoral BMD were carried out using dual-energy X-ray absorptiometry (DXA) (Discovery DXA System, Hologic Inc., Bedford, MA, USA). Written informed consent was obtained from all participants. A code was assigned to each woman at the beginning of the trial which was used to track the data throughout the study. The inclusion criteria were women with osteopenia (T-score <−1 and >−2.5) and more than 5 years post-menopause. The risk of fracture was evaluated by using the FRAX^®^ tool [10] based on an algorithm that takes into account demographic characteristics, medical history, some clinical risk factors as well as bone mineral density (BMD) at the femoral neck. The exclusion criteria were risk of fracture (FRAX: >20 major osteoporotic fractures; >3 hip fractures), hyperkalemia, renal insufficiency, urolithiasis, use of therapies influencing bone metabolism (e.g., corticosteroids, aromatase inhibitors and estrogens), use of drugs (such as diuretics and proton pump inhibitors), calcium and potassium supplements, vitamin D, current or recent (less than three years prior to the start of the study) use of bisphosphonates, gastrointestinal disorders which hamper nutrient absorption, and mental or psychiatric disorders which precluded the possibility of correctly adhering to the protocol. All the women were interviewed regarding food habits; they followed their usual diet throughout the duration of the study. Participants were assigned to the treatment or the placebo group using a randomization sequence created by independent personnel (IOR pharmacy) using a free online tool (http://randomization.com) and were stratified with a 1:1 allocation using a random block size of 4.

### 2.2. Supplements

The K citrate and the placebo were kindly provided by a manufacturer (Biohealth, Turin, Italy). They were in tablet form, identical in appearance, and were packaged in bottles which were rendered anonymous (A and B) until the end of the study. The products were stored at the IOR pharmacy and dispensed according to the computer-generated randomization list. Each woman received the supplements in the corresponding prepacked bottle. Both the subjects and the investigators who followed up the participants were blinded to group assignment. All subjects had daily supplementation with vitamin D3 cholecalciferol (400 IU day^−1^) and calcium carbonate (500 mg day^−1^). The participants assigned to the experimental group received 30 mEq K citrate daily in two tablets for oral administration; those assigned to the placebo group received the same quantity of tablets containing only the excipients. The participants had clinic visits at the time of randomization (baseline), and at 3 and 6 months. At each time point, they underwent blood and urine evaluations. The laboratory staff performing the assays was unaware of the source of the samples which were identified by numeric codes.

### 2.3. Blood Analyses

Fasting venous blood samples were collected and analyzed for creatinine, sodium, potassium, total calcium, inorganic phosphate, parathyroid hormone (PTH), and 1,25-dihydroxyvitamin D. These analytes were measured by routine methods.

### 2.4. Urine Analyses

Urine samples were collected using a standardized protocol (Lithotest^®^, Biohealth, Turin, Italy), and the metabolic profile was examined in an external certified lab (Lithocenter, Turin, Italy). Briefly, during collection, 24-hour (24 h) urine was aliquoted into two fractions to which 10 mL hydrochloric acid (to prevent calcium/phosphate precipitation) and 1 mL Hibitane (to prevent bacterial contamination) were added. The acidified aliquot was used to quantify calcium (Ca), citrate, magnesium (Mg), oxalate, phosphate (PO4) and sulphate (SO_4_); the Hibitane aliquot was tested for ammonium, chloride (Cl), creatinine, pH, potassium (K), sodium (Na), urea, and urate. In addition, a sample of fasting-morning urine was collected within 2 h after the 24 h collection ended, and was analyzed for citrate, calcium, urate (all expressed as creatinine ratios) and pH. Reference values were established on the basis of the assessment of mineral metabolic profile to evaluate the lithogenic risk [28,29,30]. The renal acid load was determined by calculating the 24 h urinary “potential renal acid load” (uPRAL) as follows [31]:

[Cl (mmol day^−1^) + SO_4_ (mmol day^−1^) × 2 + PO_4_ (mmol day^−1^) × 1.8] − [Na (mmol day^−1^) + K (mmol day^−1^) + Mg (mmol day^−1^) × 2 + Ca (mmol day^−1^) × 2]

### 2.5. Immunoenzymatic Assay of BTM

The serum level of the bone turnover markers (BTMs) was evaluated at baseline and after 3 and 6 months of treatment. The BTM assay was carried out by following the recommended actions to minimize the pre-analytical variability [32]. The blood samples were collected after overnight fasting (between 08:00 and 10:00 a.m.), centrifuged within 2 h from collection, at 1000 g min^−1^ for 15 min and at room temperature. The serum was transferred into cryotubes (0.5 mL of serum per cryotube) and frozen at −80 °C, within 1 h after centrifugation, until testing. The BTM serum levels were measured using commercially available reagents based on a sandwich enzyme immunoassay technique, following the manufacturer’s protocols, with each sample tested in duplicate. The selected BTMs were related to (i) the number of osteoclasts (tartrate-resistant acid phosphatase 5b; TRACP5b); (ii) osteoblast function (bone alkaline phosphatase; BAP); (iii) type I collagen degradation (carboxy-terminal telopeptide; CTX); (iv) type I collagen precursor (procollagen type 1 N terminal propeptide; PINP). Source and technical notes of reagents used in this study are shown in Table S1.

### 2.6. Calculations and Statistical Analysis

All calculations and analyses were carried out using StatView 5.01 for Windows (SAS Institute Inc., Cary, NC, USA) and MedCalc Statistical Software version 18.2.1 (MedCalc Software bvba, Ostend, Belgium). The sample size calculation was based on a previous randomized clinical trial (RCT) aimed to evaluate the effect of K citrate on calcium metabolism and BTM in a cohort of individuals (males and females, age >55 years) treated for 6 months [33]. The present RCT was powered considering a Type I error (α) = 0.05 (two-sided), and Type II error (β) = 0.20 (power is 80%). Fifteen patients per group were sufficient, but the expected proportion of dropouts was 20%; therefore, as a precaution, 20 women in each group were recruited. Quantitative data were expressed as the arithmetic mean plus or minus the standard error of the mean (mean ± SEM). The D’Agostino-Pearson method was used to test the normality assumption of continuous variables, and a *log* transformation was applied when the data distribution was non-normal. The data analysis was carried out following intention-to-treat principles. All follow-up parameters were analyzed using a “last value carried forward” approach under the intention-to-treat principle in order to address missing data [34]. The effect of K citrate on BTMs and blood/urine analytes was evaluated by applying the *t*-test, with comparison within the groups (paired analysis of the effect of treatment over time), and between the groups (independent comparison between K citrate and the placebo at the same time point). In order to highlight the changes over time, the results were expressed in line-graphs as deviations from the value observed before treatment, i.e.,

[(BTM concentration at time point × BTM concentration at baseline^−1^) − 1]

Thus, obtaining a baseline value equal to zero. The degree of association between the continuous variables was calculated by measuring the Pearson correlation coefficient. Differences and correlations were considered to be statistically significant when the *p* value was <0.05.

## 3. Results

### 3.1. Demographic and Clinical Characteristics of the Participants at Baseline

**Figure 1:**
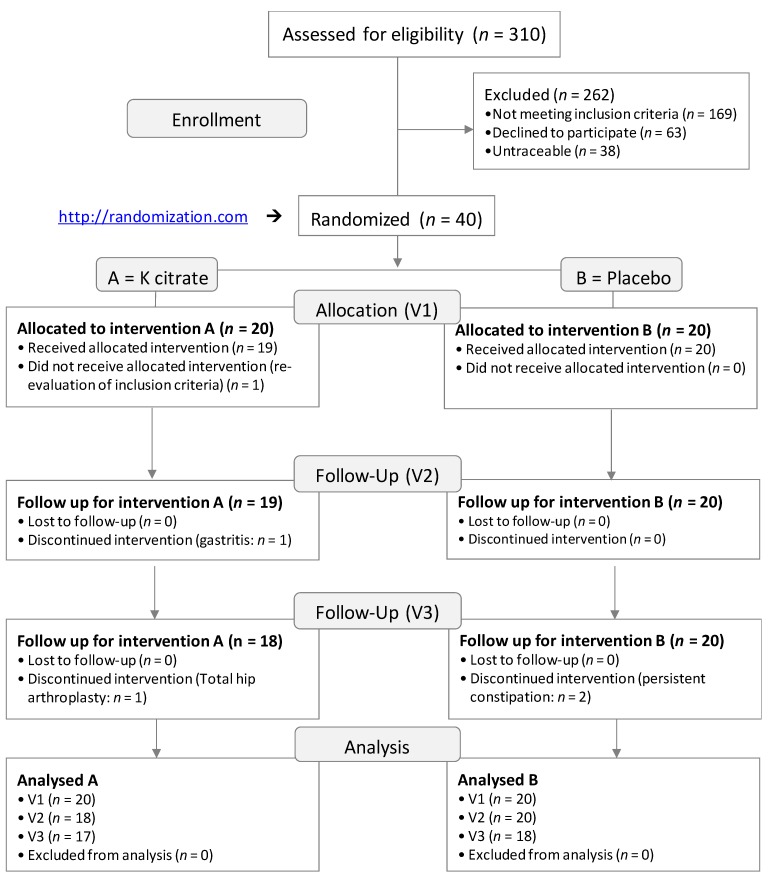
CONSORT flow diagram.

From January 2016 to June 2017, 310 women with osteopenia and more than 5 years post-menopause were screened. Forty women met the inclusion criteria and expressed their consent to be enrolled in the randomized controlled trial (RCT). The flow diagram in Figure 1 shows the numbers of participants who were randomly assigned to each group, received the intended treatment, and were analyzed for the primary outcome. Simultaneously, the losses and exclusions after randomization, together with their reasons, have been displayed [35]. Seventeen out of 20 patients in Group A (K citrate) and 18/20 in Group B (placebo) completed the study. The dropouts related to the interventions were 1/20 in the K citrate group (gastritis) and 2/20 in the placebo group (persistent constipation). Two patients decided to discontinue treatment for personal reasons.

Adherence to the treatment was 90.3 ± 9% for the K citrate group and 91.2 ± 10% for the placebo group. All women were interviewed about food habits and they declared to consume a non-vegetarian diet, with a low to moderate amount of animal protein, while fruits and plant food was more abundant. The FRAX score did not vary during the follow-up, since relevant events that could influence the algorithm and modify the risk of fracture were not recorded. At baseline, the demographic and clinical characteristics of the two groups were comparable, including age, postmenopausal years, body mass index (BMI), femur and lumbar T-scores and risk for osteoporotic fractures (Table 1). Similarly, no significant differences were found in creatinine, sodium, potassium, total calcium, inorganic phosphate, magnesium, parathyroid hormone and 1,25-dihydroxyvitamin (Table 2). Regarding the urinary metabolic profile, the only difference at baseline was the excretion of oxalate in the 24 h urine (Table 3). However, oxalate excretion was not a target variable of the present study. The circulating levels of BTMs at baseline were comparable and no significant difference between the groups was observed (Table 4).

### 3.2. K Citrate Supplementation and Changes in Urinary Parameters

The urine metabolic profile was interpreted on the basis of the reference values used to assess the mineral metabolism [19,20,21,36]. At baseline, it was found that potassium excretion was <50 mEq in 90% of the 24 h urine samples, and 85% of women exhibited alteration in pH and/or citrate excretion. In particular, in 24 h urine a low pH (<5.5) was found in 22.5% of cases, a low total citrate excretion (≤3.3 mmol day^−1^) in 57.5%, and a low citrate/creatinine ratio (≤0.3 mmol mol^−1^) in 32.5%. In fasting-morning urine, low pH was observed in 35% of patients, low citrate concentration (≤3.3 mmol L^−1^) in 62.5%, and a low ratio citrate/creatinine in 25%. The uPRAL at baseline was negative in 37/40 patients (range −107.5 to 23). The pH value of fasting-morning urine correlated directly and significantly with potassium excretion (*R* = 0.33, *p* = 0.02) but not with 24 h citrate levels (*R* = 0.09; *p* = 0.58) while a highly significant correlation between potassium and citrate excretion was found (*R* = 0.47, *p* = 0.002). As shown in Table 3, the group treated with K citrate for 6 months exhibited potassium excretion significantly higher in comparison to the placebo group; pH and citrate excretion were also significantly more elevated in the experimental group in both fasting-morning and 24 h urine.

**Figure 2:**
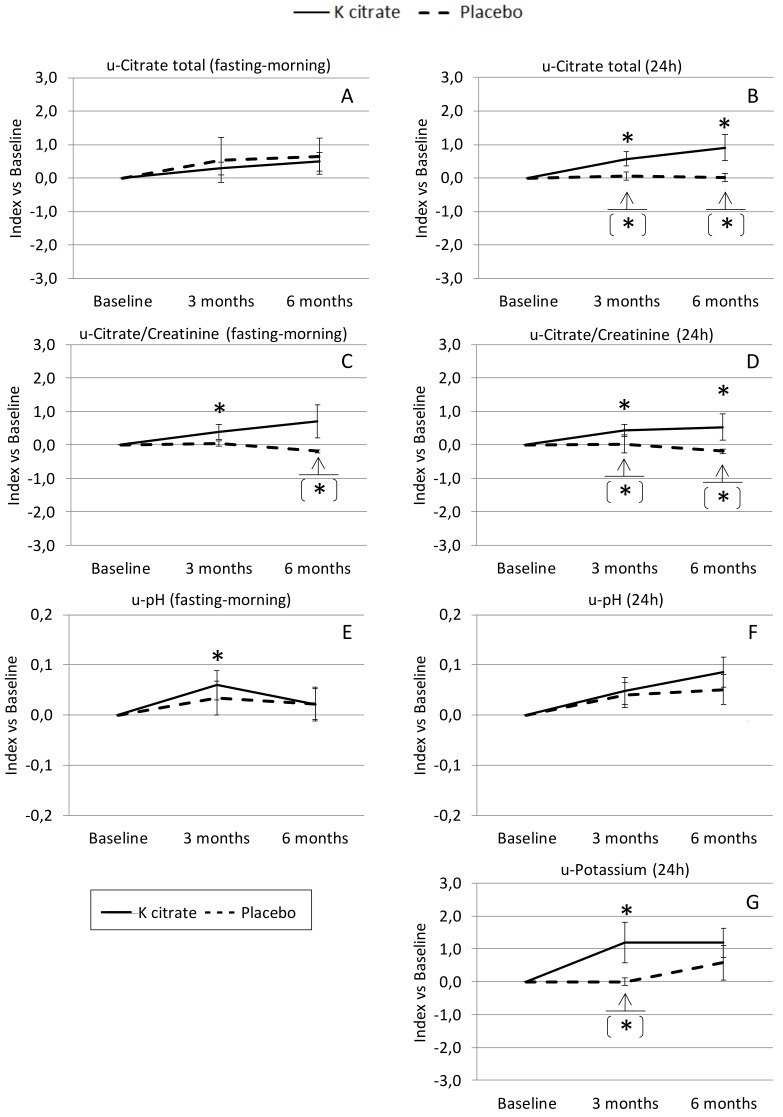
Changes in urine (u) parameters according to treatment group. The line-graphs highlight the changes observed in both fasting-morning urine (**A**,**C**,**E**) and 24 h urine (**B**,**D**,**F**,**G**). Data are expressed as mean plus or minus the standard error of the mean (mean ± SEM) of the variations over time calculated in each subject as [(concentration at time point × concentration at baseline^−1^) − 1], with the baseline = 0. Symbols indicate the statistically significant differences over time (paired *t* test; * = *p* < 0.05 vs. Baseline); the symbol in square brackets indicates a statistically significant difference between groups at the time point highlighted by the arrow (unpaired *t* test; * = *p* < 0.05).

In order to minimize individual variability, the changes over time were analyzed, calculating the deviation from the value observed at baseline (Figure 2). The urinary excretion of citrate and potassium changed significantly only in the women treated with K citrate, and significant differences in comparison to the placebo group were already observed at 3 months. An increase in urine pH was found in both patient groups, even if the effect over time was significant only after K citrate supplementation.

### 3.3. K Citrate Supplementation and Changes in BTM

**Figure 3:**
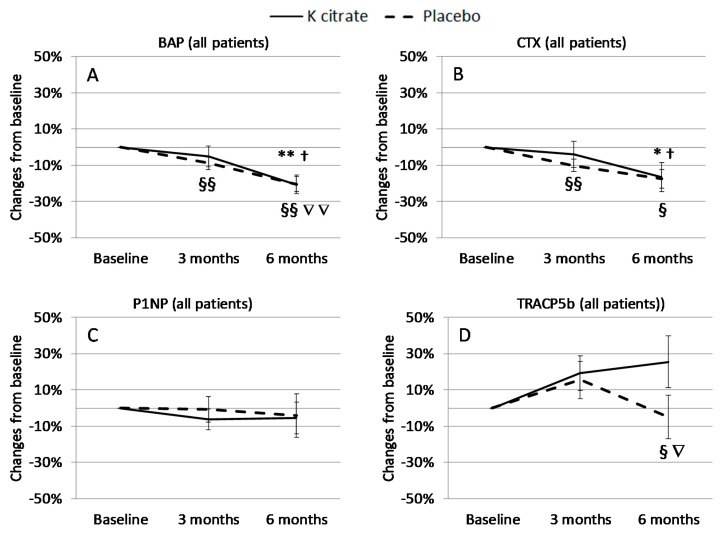
Bone turnover marker (BTM) changes (%) according to treatment group. The data regarding bone alkaline phosphatase (BAP) (**A**), carboxy-terminal telopeptide of type I collagen (CTX) (**B**), procollagen type 1 N terminal propeptide (PINP) (**C**) and tartrate-resistant acid phosphatase 5b (TRACP5b) (**D**) are expressed as mean ± SEM of the changes calculated in each subject as [(BTM concentration at the time point × BTM concentration at baseline^−1^) − 1] × 100, where the baseline is 0. Symbols indicate the statistically significant values obtained from the paired *t* test. K citrate group: * = *p* < 0.05 vs. Baseline; ** = *p* < 0.005 vs. Baseline; † = *p* < 0.05 vs. 3 months. Placebo group: § = *p* < 0.05 vs. Baseline; §§ = *p* < 0.005 vs. Baseline; ∇∇ = *p* < 0.005 vs. 3 months.

The serum levels of the BTMs are shown in Table 4, but no significant difference between K citrate and the placebo was found at baseline and the following time points. Patient-by-patient analysis showed that CTX and BAP decreased significantly in both groups (Figure 3), thus, suggesting that K citrate supplementation does not improve the beneficial effects of treatment with calcium and vitamin D. Changes in the PINP levels were largely variable while, unexpectedly, TRACP5b, a marker related to OC number and activity, tended to increase after treatment with K citrate.

**Figure 4:**
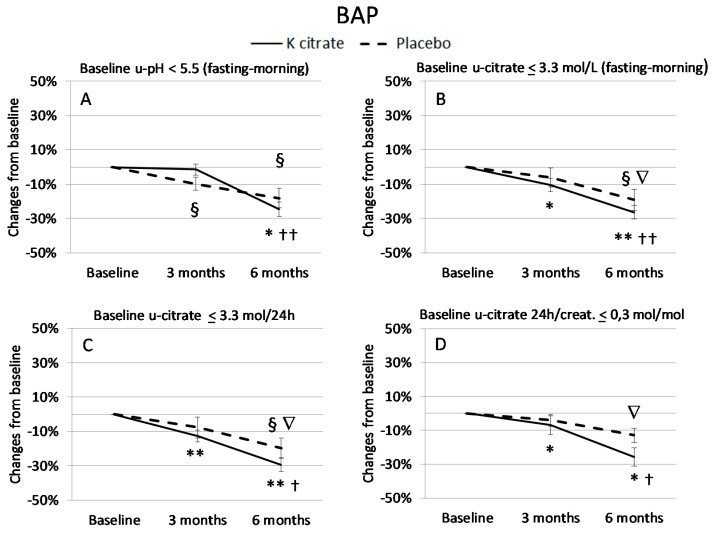
BAP changes (%) in the subgroups of patients with signs of low-grade acidosis at baseline. The BAP changes were analyzed in women who exhibited low urinary (u) pH in fasting-morning urine (**A**: *n* = 14), and low citrate excretion in both fasting-morning urine (**B**: *n* = 25) and 24 h urine (**C:** *n* = 23; **D**: *n* = 13). Data are expressed as mean ± SEM of the BAP change calculated in each subject as described in Figure 3. The symbols indicate the statistically significant values obtained with the paired *t* test. K citrate group: * = *p* < 0.05 vs. Baseline; ** = *p* < 0.005 vs. Baseline; † = *p* < 0.05 vs. 3 months; †† = *p* < 0.005 vs. 3 months. Placebo group: § = *p*< 0.05 vs. Baseline; ∇ = *p* < 0.05 vs. 3 months.

**Figure 5:**
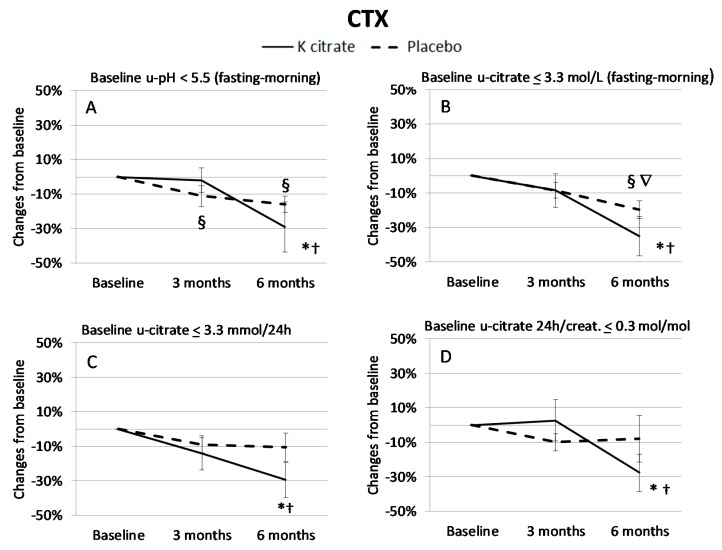
CTX changes (%) in subgroups of patients with signs of low-grade acidosis at baseline. The CTX changes were analyzed in the women who exhibited low urinary (u) pH in fasting-morning urine (**A**: *n* = 14), and low citrate excretion in both fasting-morning urine (**B**: *n* = 25) and 24 h urine (**C**: *n* = 23; **D**: *n* = 13). Data are expressed as mean ± SEM of the CTX change calculated in each subject as described in Figure 3. The symbols indicate the statistically significant values obtained with the paired *t* test. K citrate group: * = *p* < 0.05 vs. Baseline; † = *p* < 0.05 vs. 3 months. Placebo group: § = *p* < 0.05 vs. Baseline; ∇ = *p* < 0.05 vs. 3 months.

In order to explore the effects of K citrate more in depth, patients who exhibited low urine pH and/or low citrate excretion before treatment were considered separately, while there was no need to identify those who had low potassium excretion since the defect was present in almost all patients. Interestingly, in subgroups there is a benefit from K citrate supplementation compared to the entire case series (Figure 4 and Figure 5). In particular, women with a low citrate excretion in 24 h urine gained benefits from the experimental treatment, resulting in a 30% mean decrease in BAP and CTX at 6 months. A significant reduction was also evident in subjects with alterations in fasting-morning urine, i.e., low citrate excretion (BAP: −25%; CTX: −35%) and low pH (BAP: −25%; CTX: −30%).

**Figure 6:**
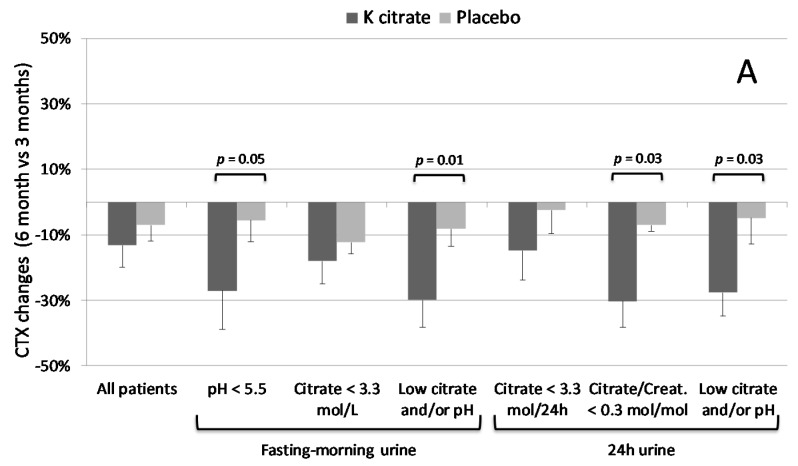

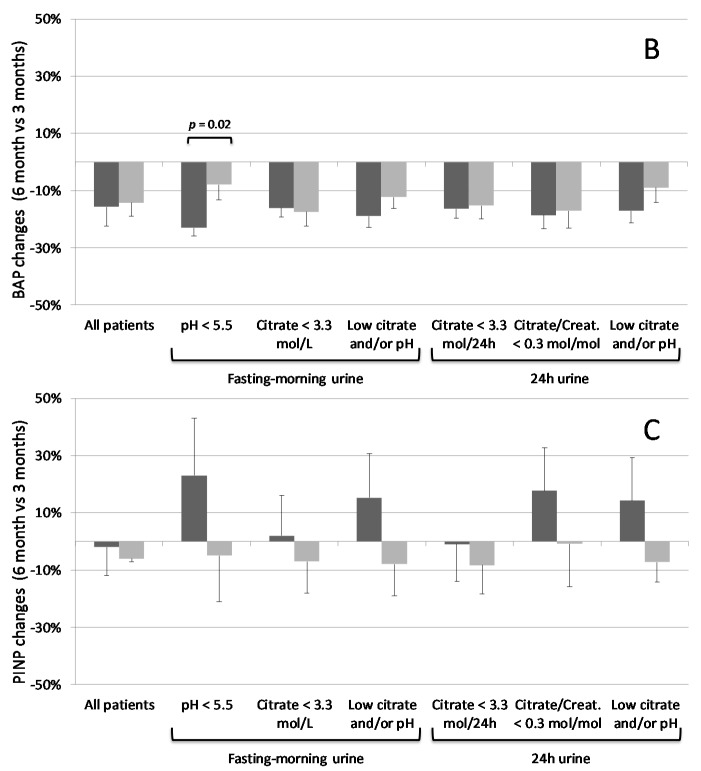
BTM changes in the last three-month period of treatment. Data are expressed as mean ± SEM of the changes calculated in each subject as [(BTM concentration at 6 months × BTM concentration at 3 months^−1^) − 1] × 100, where the 3-month value is 0. The bars represent the variation in CTX (**A**), BAP (**B**), and PINP (**C**) in all patients and subgroups who completed the follow-up. All patients: *n* = 35. Number of patients with altered parameters in fasting-morning urine: *n* = 13, pH <5.5; *n* = 20, citrate <3.3 mol L^−1^; *n* = 32, low citrate and/or pH. Number of patients with altered parameters in 24 h urine: *n* = 20, citrate <3.3 mol day^−1^; *n* = 11, citrate/creatinine <0.3 mol mol^−1^; *n* = 32, citrate and/or pH. The significant differences between K citrate and the placebo are highlighted by *p* values < 0.05 over the bars.

In particular, the changes induced by K citrate on CTX were more evident from three to six months, and statistically significant differences were detectable in subgroups with low pH and low citrate excretion before treatment (Figure 6A). The differences were more pronounced when the osteopenic women who exhibited at least one of the above defects were considered in a separate subgroup. The changes in BAP in the last three-month period were significantly different only in subjects with a low pH in fasting-morning urine (Figure 6B). Interestingly, PINP levels increased in the patients with low pH and low citrate excretion who were treated with K citrate while they decreased in the placebo group; however, the high variability of the data did not allow pointing out the significant differences (Figure 6C). No notable results were found for TRACP5b (data not shown). Concerning the relationship between urine parameters and BTMs, it was found that PINP correlated significantly with 24 h citrate excretion in the entire population (*R* = 0.24, *p* = 0.003) and with pH values in the subgroup with low pH in fasting-morning urine (*R* = 0.31; *p* = 0.003).

## 4. Discussion

The relationship involving acid-base disequilibrium, mineral metabolism and bone health status has previously been reported [15,17,18,19,20,21]; however, the benefits of targeting the acid overload to prevent bone loss have not yet been established and, at present, the indications for alkalizing supplementation have to be fully elucidated [25]. This study evaluated whether K citrate supplementation influenced bone turnover by modifying the serum markers, as would be expected by treatments which oppose bone loss progression. To highlight the conditions in which dietary supplements could be recommended, particular attention was paid to the metabolic features of the patients who gained major benefits in terms of BTM changes.

The mineral metabolic profile of patients was delineated by testing urine parameters which, in previous reports, were seen to have significant implications for monitoring bone health status [20,21,22]. The present study found that potassium and citrate excretion was below the threshold value in a considerable proportion of subjects, and, in a smaller group, even low urine pH was found, especially in fasting-morning urine. These alterations were consistent with low-grade metabolic acidosis which promoted bone loss and favored precipitation of the calcium salts [22,37,38].

While it was expected that daily supplementation with K citrate would be able to normalize the urine defects, favorable results on BTM serum levels were more uncertain since the benefits of this supplementation in preventing bone loss are still unclear.

By analyzing the entire case series, the present study did not observe differences between the experimental and the placebo group, indicating that, in order to prevent bone loss progression, it was not worth using K citrate indiscriminately in all osteopenic women since it did not add anything to the benefits induced by calcium and vitamin D supplements.

Previous randomized clinical trials have shown that K citrate is able to reduce circulating BTM levels over time [29,33,39,40,41,42,43,44,45], but the results were not homogeneous and significant differences between experimental treatment and a placebo have been found in only a few studies and for a limited number of markers [33,42,43,44]. The heterogeneity of the above results mainly depended on marked differences in study design and inclusion criteria of study populations, leading to the hypothesis that K citrate could be more effective in some subjects while others have no benefits.

In the present study, it was assumed that the effects of the alkaline supplementation with K citrate on bone turnover would be more significant when the signs of low-grade acidosis were identified before treatment, i.e., when the specific target was present. The hypothesis was confirmed since the subjects with low urine pH and/or low citrate excretion were analyzed separately and, interestingly, the decrease in BAP and CTX was more pronounced after K citrate supplementation compared to calcium and vitamin D alone. Surprisingly, the BTM changes were similar to those observed after the administration of antiresorptive drugs while they were unexpected after nonpharmacological treatment. This is a noteworthy result since it has been recognized that a CTX reduction greater than 30% has been correlated with a significant decrease in the incidence of osteoporotic fracture incidence [32].

The BTMs selected in this study cover different stages of bone remodeling. PINP reflects the production of type I collagen by OBs and the highest concentration is found during the proliferative phase; BAP is mainly expressed during matrix maturation; TRACP5b is related to the OC number while CTX is indicative of their activity [46]. In general, BTM levels reflect the increased BRU activity and correlate with the bone loss [32] and, therefore, they are considered a useful tool for assessing the response to anti-osteoporotic drugs. CTX and PINP are recommended by international committees as the reference markers for bone resorption and bone formation, respectively [47], while data regarding the clinical usefulness of BAP and TRACP5b are not always consistent [32]. Both resorption and formation markers decrease due to the effect of antiresorptive therapy, since the decrease in OC number and activity leads to a rapid reduction in bone formation by OBs determined by the lack of coupling factors [32]. Conversely, the use of anabolic agents, e.g., teriparatide, induces a greater and more rapid increase in bone formation markers after 6 months of treatment, especially PINP [48], but BAP and CTX may also increase over a longer time frame [49]. In this study, it was shown that BAP and CTX decrease, as expected, after interventions which oppose bone loss progression, but a variable increase in PINP and TRACP5b levels which is more difficult to interpret was also observed.

The above results allowed hypothesizing the dual activity of K citrate, similar to that of strontium ranelate which reduced bone resorption and increased bone formation [50]. First, alkali supplementation counteracted the pro-osteoclastogenic effect of the acid-base disequilibrium, thus, mimicking the pharmacological properties of antiresorptive drugs. Subsequently, when the citrate and potassium levels were normalized, and the acid-base balance was restored, K citrate could have exerted an anabolic effect, as suggested by the PINP increase observed in the last three-month period. It can be hypothesized that, if the activity of bone formation was favored, new OC would also be formed in order to maintain bone homeostasis, and the increase in TRACP5b level could have reflected the growing OC number. An increase in PINP levels after K citrate supplementation has also been described by Jehle et al. [44] while, to our knowledge, TRACP5b has not been investigated in clinical trials regarding the use of K citrate in osteopenic women.

The low excretion of potassium and citrate as well as the low pH that we observed in our case series are signs of low-grade acidosis that could be related to consumption of a high acid-generating diet since it is recognized that some foods have a higher acid content than others [5,51]. However, all the women were interviewed regarding food habits; they reported consuming a low/moderate amount of animal protein and abundant plant food [14,52]. The scarce dietary acid load was proven by the low daily excretion of some nutritional markers (e.g., creatinine, sulphate, phosphate and urea) and also confirmed by uPRAL, i.e., the daily net acid excretion without its organic anion component which had a negative value in more than 90% of the patients [31]. In agreement with the results of other authors, a direct linkage involving food, low-grade acidosis and osteopenia did not seem to be plausible in women who consumed a Mediterranean diet [51,53], and there are probably other causes which played a pivotal role in determining both urine changes and bone weakening [54].

Low potassium excretion was the most frequent urine alteration in the present case series, and it could have been the earliest change which, in turn, influenced the others. It is known that potassium depletion decreases intracellular pH and increases H+ secretion into the tubular lumen, causing hypocitraturia and low urine pH [38]. The present study confirmed that the lower the potassium excretion, the lower the citrate excretion, and, in turn, the lower the pH value. Since hypokalemia was never found in the cohort of osteopenic women, the low potassium excretion suggested that the majority of the subjects had to conserve this alkali element to maintain serum levels in a normal range and to ensure physiological demand.

**Figure 7:**
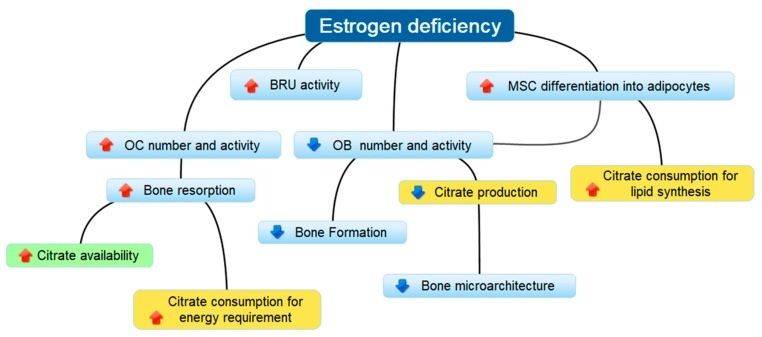
Diagram showing some of the mechanisms that explain the relationship between diminished citrate excretion and bone loss in postmenopausal women. Estrogen deficiency promotes the activity of multicellular units (BRUs) containing osteoclasts (OCs) and osteoblasts (OBs) which, in sequence, resorb old bone and form new bone. The “net bone loss” depends on the increased bone resorption by OCs which exceed the new bone formation since osteogenic precursors (MSCs) are destined to differentiate into adipocytes, and the number and function of OBs are decreased. Bone resorption should favor the availability of citrate embedded in the bone matrix (green topic), but the lower production and the higher consumption (yellow topics) cause a “net citrate loss”.

Other than low potassium excretion, there were additional reasons which could have explained the low citraturia in postmenopausal osteopenia. It is well known that estrogen deficiency and aging are, for the most part, responsible for the depletion of bone mass [3], but they are also involved in the lowering of citrate excretion [54,55]. True hypocitraturia is usually fixed as lower than 320 mg day^−1^ (or 1.7 mmol day^−1^) and is a common finding in recurrent calcium stone formers; however, low citrate excretion, less severe, has been described in healthy individuals [38], postmenopausal women [55] and in subjects with a low bone mass [20,21]. Postmenopausal estrogenic decline influences the activation rate of BRUs, a multicellular whole comprising of OCs and OBs which sequentially resorb old bone and form new bone. Nevertheless, bone resorption increases by 90% while bone formation increases by only 45%, thus, resulting in a “net bone loss” [56]. This gap depends on the effects that the lack of estrogen has on bone cells. On one hand, the activity of the receptor activator of the nuclear factor-κ B ligand (RANKL), a key factor in OC differentiation, is promoted; on the other hand, the osteogenic differentiation of mesenchymal stem cells (MSCs) and the survival of mature OBs is suppressed [3]. Since mature OBs are highly specialized cells capable of synthesizing citrate which is necessary for the stability of hydroxyapatite nanocrystal/collagen complexes, the lower citrate production due to the impaired OB function accompanies the disruption of the bone microarchitecture [57]. Moreover, OC differentiation and bone resorption are energy-demanding processes, and the citrate which is synthesized cannot be accumulated because it is essentially utilized through the citric acid cycle [58,59]. Similarly, the MSC differentiation towards adipocytes requires more citrate as a source of cytosolic acetyl-CoA for lipid biosynthesis [60]. As a final result, the estrogen deficiency leads to a “net citrate loss” which could explain the diminished citrate excretion observed in postmenopausal women. The mechanisms by which estrogenic hormones influence citrate availability are summarized in Figure 7.

Even though the aims of the present study were reached, there were some limitations. First of all, the sample size has been calculated empirically on the basis of a previous report that tested the hypothesis of K citrate superiority without considering the influence of unexpected variables. Instead, the beneficial effects of K citrate were detected only in osteopenic women with specific metabolic defects, and, as a consequence, the probability of finding statistically significant results was decreased since the number of cases within the subgroups was lower [61]. Second, the exclusion of a variety of possible confounding factors did not allow generalizing the results since the authors did not know whether concomitant diseases and related treatments could have interfered with the K citrate activity. Finally, the short follow-up did not allow the verification as to whether the significant BTM changes corresponded to an actual increase in BMD.

## 5. Conclusions

This study showed that signs of low-grade acidosis, including low urine pH and low excretion of potassium and citrate, were detectable in a considerable proportion of osteopenic women. In this specific subgroup, K citrate supplementation induced positive effects on bone turnover while its indiscriminate use is not advisable since it does not add anything to the benefits induced by calcium and vitamin D. These findings suggest that K citrate could be recommended to prevent bone loss in postmenopausal osteopenia, but the presence of a low-grade acidosis should be documented before treatment. The promising results of the pilot trial have to be confirmed in a larger case series and over a longer time period.

## Figures and Tables

**Table 1:** Demographic characteristics of the patients analyzed ^1^.

	K Citrate (*n* = 20)	Placebo (*n* = 20)
Age	60.8 ± 1.0 (52.0–69.0; 62.0)	58.2 ± 1.1 (48.0–70.0; 57.0)
Years post-menopause	11.5 ± 1.4 (5.0–31.0; 9.0)	8.3 ± 0.9 (5.0–20.0; 7.5)
BMI (kg m^−2^)	23.7 ± 1.0 (18.7–37.0; 22.7)	22.9 ± 0.8 (18.3–31.2; 21.8)
*T*-score		
Femoral neck L2–L4	−1.6 ± 0.1 (−2.4 to −0.6; −1.7) −1.7 ± 0.1 (−2.3 to −0.6; −1.9)	−1.7 ± 0.1 (−2.4 to −0.5; −1.8) −1.5 ± 0.1 (−2.4 to − 0.1; −1.5)
FRAX		
Major osteoporotic risk Minor osteoporotic risk	5.7 ± 0.8 (2.2–19.0; 4.9) 1.1 ± 0.2 (0–3.1; 0.9)	4.8 ± 0.3 (2.4–8.6; 4.7) 1.0 ± 0.1 (0.1–1.9; 1.0)

^1^ Data expressed as mean plus or minus the standard error of the mean (mean ± SEM) (min–max; median).

**Table 2:** Chemistry and hormones levels in serum ^1^.

Analyte (Unit)	Reference Values	Baseline		3 Months		6 Months	
		K Citrate	Placebo	K Citrate	Placebo	K Citrate	Placebo
Creatinine (mg dL^−1^)	0.5–1.2	0.7 ± 0.0	0.7 ± 0.0	0.7 ± 0.0	0.8 ± 0.0	0.8 ± 0.0	0.7 ± 0.0
Calcium (mg dL^−1^)	8.6–10.5	9.6 ± 0.1	9.6 ± 0.1	9.5 ± 0.1	9.6 ± 0.1	9.6 ± 0.1	9.7 ± 0.1
Phosphorus (mg dL^−1^)	2.5–4.5	3.6 ± 0.1	3.7 ± 0.1	3.7 ± 0.1	3.8 ± 0.1	3.7 ± 0.1	3.8 ± 0.1
Magnesium (mg dL^−1^)	1.6–2.6	2.2 ± 0.0	2.1 ± 0.0	2.2 ± 0.0	2.1 ± 0.0	2.2 ± 0.0	2.2 ± 0.0
Sodium (mg dL^−1^)	136.0–145.0	142.2 ± 0.5	142.3 ± 0.4	141.2 ± 0.5	141.4 ± 0.5	140.1 ± 0.6	140.6 ± 0.5
Potassium (mg dl^−1^)	3.5–5.3	4.5 ± 0.1	4.5 ± 0.1	4.5 ± 0.1	4.4 ± 0.1	4.4 ± 0.1	4.4 ± 0.1
PTH (pg mL^−1^)	12.0–88.0	46.5 ± 3.9	47.0 ± 3.3	48.0 ± 4.6	44.2 ± 2.6	44.2 ± 4.9	47.0 ± 4.3
25OH Vitamin D3 (ng mL^−1^)	<20 failure 20–100 sufficiency >100 potential toxicity	31.6 ± 2.3	32.1 ± 1.6	31.0 ± 2.4	34.1 ± 1.9	32.1 ± 1.9	34.9 ± 1.3

^1^ Data expressed as mean ± SEM; *p* value for *t* tests (unpaired comparison) were not significant.

**Table 3:** Metabolic profile of the participants evaluated using 24 h and fasting-morning urine samples ^1^.

Analyte (Unit)	Reference Values	Baseline		3 Months		6 Months	
		**K Citrate**	**Placebo**	**K Citrate**	**Placebo**	**K Citrate**	**Placebo**
**24 h urine**							
Diuresis (mL)		1724.0 ± 155.0	1702.0 ± 150.0	1763.0 ± 181.0	1721.0 ± 163.0	1899.0 ± 179.0	1853.0 ± 192.0
pH	5.5–7	6.1 ± 0.1	6.0 ± 0.2	6.5 ± 0.2	6.3 ± 0.1	6.6 ± 0.1	6.2 ± 0.1 ^a^
Creatinine (g day^−1^)	1.3–1.8	0.9 ± 0.1	0.9 ± 0.1	1.0 ± 0.1	0.8 ± 0.1	1.0 ± 0.1	1.0 ± 0.1
Citrate (mmol day^−1^)	>3.3	3.0 ± 0.4	3.5 ± 0.4	4.0 ± 0.5	3.2 ± 0.3	4.0 ± 0.4	3.2 ± 0.3
Citrate/Creatinine (mol mol^−^^1^)	>0.3	0.4 ± 0.1	0.4 ± 0.0	0.5 ± 0.1	0.5 ± 0.0	0.5 ± 0.0	0.4 ± 0.0 ^a^
Potassium (mEq day^−^^1^)	50–100	28.6 ± 3.4	30.0 ± 2.9	43.0 ± 10.6	28.5 ± 6.6	46.6 ± 4.4	35.4 ± 3.4 ^a^
Calcium (mg day^−^^1^)	<200	117.4 ± 16.3	128.7 ± 12.4	124.4 ± 18.3	123.2 ± 13.4	142.8 ± 17.8	140.9 ± 15.5
Calcium/Creatinine (mg mg^−^^1^)	0.02–0.2	0.2 ± 0.0	0.2 ± 0.0	0.1 ± 0.0	0.2 ± 0.0	0.2 ± 0.0	0.2 ± 0.0
Oxalate (mmol day^−^^1^)	<0.5	0.3 ± 0.0	0.3 ± 0.0	0.3 ± 0.0	0.3 ± 0.0	0.3 ± 0.0	0.3 ± 0.0
Oxalate/Creatinine (mmol mol^−^^1^)	<50	48.9 ± 3.1	35.2 ± 2.7 ^b^	43.0 ± 4.1	40.8 ± 4.5	33.8 ± 1.9	33.9 ± 2.9
Ammonium (mmol day^−^^1^)	20–50	20.7 ± 1.0	21.2 ± 0.8	23.3 ± 2.0	20.5 ± 0.7	23.2 ± 1.1	22.1 ± 1.1
Chloride (mEq day^−^^1^)	140–240	121.0 ± 11.4	124.1 ± 6.6	180.8 ± 7.2	126.3 ± 9.1	100.9 ± 8.3	110.5 ± 9.8
Magnesium (mg day^−^^1^)	>50	58.2 ± 5.7	59.1 ± 4.5	61.6 ± 7.6	54.1 ± 5.3	87.5 ± 13.5	70.7 ± 12.9
Phosphate (mmol day^−^^1^)	<42	13.0 ± 1.2	12.1 ± 1.0	15.1± 3.1	10.0 ± 0.7	12.9 ± 1.7	9.8 ± 0.7
Sodium (mEq day^−^^1^)	50–200	101.5 ± 9.8	99.5 ± 5.0	97.9 ± 8.1	111.8 ± 8.5	98.8 ± 8.5	107.2 ± 10.7
Sulphate (mmol day^−^^1^)	6–30	11.6 ± 1.0	12.4 ± 0.9	13.8 ± 2.6	11.8 ± 0.7	13.0 ± 1.4	12.3 ± 0.9
Urea (g day^−^^1^)	10–35	15.4 ± 0.9	8.5 ± 1.3	17.4 ± 2.0	15.5 ± 0.7	17.2 ± 1.3	15.8 ± 1.3
Uric Acid (mg day^−^^1^)	<600	417.6 ± 25.0	413.5 ± 20.6	499.8 ± 46.0	389.7 ± 19.0 ^a^	461.4 ± 32.9	395.5 ± 25.1
**Fasting-morning urine**							
pH	5.5–7	6.1 ± 0.2	5.9 ± 0.2	6.3 ± 0.2	6.0 ± 0.2	6.1 ± 0.1	5.6 ± 0.2 ^a^
Creatinine (mg dL^−^^1^)	n.d.^2^	76.4 ± 9.6	83.1 ± 12.7	78.4 ± 10.4	63.0 ± 7.7	81.5 ± 11.1	97.82± 15.3
Citrate (mmol L^−^^1^)	>3.3	2.8 ± 0.3	3.4 ± 0.5	3.4 ± 0.5	2.5 ± 0.3	3.5 ± 0.5	3.3 ± 0.5
Citrate/Creatinine (mol mol^−^^1^)	>0.3	0.5 ± 0.0	0.5 ± 0.0	0.5 ± 0.1	0.5 ± 0.0	0.5 ± 0.1	0.40 ± 0.04 ^a^
Calcium/Creatinine (mg mg^−^^1^)	0.02–0.2	0.1 ± 0.0	0.2 ± 0.0	0.1 ± 0.0	0.2 ± 0.0	0.1 ± 0.0	0.12 ± 0.02
Uric Acid (mg dL^−^^1^)	n.d. ^2^	36.7 ± 3.3	37.0 ± 4.0	39.3 ± 4.7	29.0 ± 3.0	35.6 ± 4.5	36.0 ± 4.0

^1^ Data expressed as mean ± SEM. ^2^ Not determined. ^a^ *p* < 0.05 and ^b^ is *p* < 0.005 (*t* test for unpaired comparison).

**Table 4:** Serum levels of bone turnover markers ^1^.

Marker	Baseline		3 Months		6 Months	
	K Citrate	Placebo	K Citrate	Placebo	K Citrate	Placebo
Carboxy-terminal telopeptide of type I collagen (CTX) (µg L^−1^)	0.64 ± 0.08	0.64 ± 0.05	0.63 ± 0.08	0.56 ± 0.05	0.53 ± 0.08	0.54 ± 0.06
Bone alkaline phosphatase (BAP) (µg L^−1^)	21.89 ± 1.67	20.36 ± 1.17	19.81 ± 1.67	18.27 ± 1.00	16.83 ± 1.37	15.79 ± 1.09
Procollagen type 1 N terminal propeptide (PINP) (pg L^−1^)	17.45 ± 1.48	18.82 ± 1.73	16.24 ± 1.60	18.39 ± 1.75	14.9 7 ± 1.51	16.77 ± 1.89
Tartrate-resistant acid phosphatase 5b (TRACP5b) (U L^−1^)	2.35 ± 0.20	2.64 ± 0.22	2.79 ± 0.27	2.85 ± 0.22	2.69 ± 0.29	2.25 ± 0.14

^1^ Data expressed as mean ± SEM; *p* value for *t* tests (unpaired comparison) were not significant.

## Supplementary Materials

The following are available online at http://www.mdpi.com/2072-6643/10/9/1293/s1, Table S1: Source of the immunoenzymatic assay kits, sensitivity, precision, least significant change (LSC) and reference values from healthy individuals.
