# Supplementary material for: Potassium Citrate Supplementation Decreases the Biochemical Markers of Bone Loss in a Group of Osteopenic Women: The Results of a Randomized, Double-Blind, Placebo-Controlled Pilot Study
Source: Nutrients. 2018 Sep 12;10(9):1293. doi: 10.3390/nu10091293 (PMC6164684; doi:10.3390/nu10091293)
Supplement: Supplementary file 1 [file nutrients-10-01293-s001.pdf]

Table S1. Source of the immunoenzymatic assay kits, sensitivity, precision, least significant change (LSC) and reference values from healthy individuals.

**Marker (Unit)**<br>**Commercial name (Source)**	**Detection limit**	**Min–Max intraassay CV (%)**	**Min–Max interassay CV (%)**	**LSC (%)**	**Mean ± SEM**<br>**Median**<br>**Min–Max**		
					Female (postmenopausal)	Female (premenopausal)	Male
CTX (µg L^{-1})<br>Serum Crosslaps (Immunodiagnostic systems Limited, Boldon, UK)	0.02	1.8–3.0	2.5–10.9	16.6	0.64 ± 0.36<br>0.44<br>0.12–1.35	0.38 ± 0.19<br>0.29<br>0.11–0.74	0.39 ± 0.19<br>0.29<br>0.12–0.75
BAP (µg L^{-1})<br>Ostase BAP (Immunodiagnostic systems Limited)	0.7	2.6–6.5	3.7–6.1	15.6	14.53 ± 4.21<br>13.2<br>8.0–22.4	9.0 ± 2.98<br>8.78<br>4.0–14.30	13.13 ± 3.8<br>12.3<br>7.0–20.1
PINP (pg L^{-1})<br>Human Procollagen I N-terminal Peptide (Cusabio Technology LLC, Aachen, Germany)	18.75	8	10	29.8	22.6 ± 1.7 [1]<br>20.1<br>15.4–40.8		
TRACP5b (U L^{-1})<br>Bone TRAP (Immunodiagnostic systems Limited)	0.5	6.0–6.6	5.8–7.2	21.1	3.19 ± 0.85	2.59 ± 0.78	3.06 ± 0.88

[1] Reference values have not been differentiated by gender and age.
